# Electronic, Dielectric, and Plasmonic Properties of Two-Dimensional Electride Materials X_2_N (X=Ca, Sr): A First-Principles Study

**DOI:** 10.1038/srep12285

**Published:** 2015-07-20

**Authors:** Shan Guan, Shengyuan A. Yang, Liyan Zhu, Junping Hu, Yugui Yao

**Affiliations:** 1 School of Physics, Beijing Institute of Technology, Beijing 100081, China; 2 Research Laboratory for Quantum Materials and EPD Pillar, Singapore University of Technology and Design, Singapore 487372, Singapore; 3 School of Physics and Electronic & Electrical Engineering, Huaiyin Normal University, Huaian 223300, China

## Abstract

Based on first-principles calculations, we systematically study the electronic, dielectric, and plasmonic properties of two-dimensional (2D) electride materials X_2_N (X = Ca, Sr). We show that both Ca_2_N and Sr_2_N are stable down to monolayer thickness. For thicknesses larger than 1-monolayer (1-ML), there are 2D anionic electron layers confined in the regions between the [X_2_N]^+^ layers. These electron layers are strongly trapped and have weak coupling between each other. As a result, for the thickness dependence of many properties such as the surface energy, work function, and dielectric function, the most dramatic change occurs when going from 1-ML to 2-ML. For both bulk and few-layer Ca_2_N and Sr_2_N, the in-plane and out-of-plane real components of their dielectric functions have different signs in an extended frequency range covering the near infrared, indicating their potential applications as indefinite media. We find that bulk Ca_2_N and Sr_2_N could support surface plasmon modes in the near infrared range. Moreover, tightly-bounded plasmon modes could exist in their few-layer structures. These modes have significantly shorter wavelengths (few tens of nanometers) compared with that of conventional noble metal materials, suggesting their great potential for plasmonic devices with much smaller dimensions.

Electrides are a special kind of ionic solids with cavity-trapped electrons serving as the anions^1^,^2^. These electrons are spatially separated from the cations in a regular crystalline array, and are not bound to any particular atom, molecule, or bond. The first crystalline electride, Cs^+^·(18-crown-6)_2_e^−^, was synthesized in 1983^3^, and several other electrides have been successfully discovered or predicted later on^4^,^5^,^6^,^7^,^8^,^9^,^10^,^11^,^12^,^13^,^14^. The properties of electrides are closely connected to the topology and geometry of the cavities which confine the anionic electrons^2^. The early examples of electride demonstrate confinement of zero-dimensional cavities or one-dimensional weakly linked channels^2^. Recently, a new type of electride with two-dimensional (2D) confinement of anionic electrons was discovered in dicalcium nitride (Ca_2_N) which has a layered structure^15^. From formal valence consideration, each unit cell of Ca_2_N should have one excess electron. Experimental measurements on properties such as the transport parameters and work functions, combined with first-principles calculations, indeed proved that there is a built-in anionic electron layer confined between the calcium layers, which agrees well with the chemical formula [Ca_2_N]^+^·e^−^15. This discovery of 2D electride materials (here 2D refers to the anionic electron confinement topology) has generated great research interest. First-principles calculations have predicted several other 2D electrides^16^,^17^, including the other two alkaline earth sub-nitrides Sr_2_N and Ba_2_N^18^. Recent theoretical work also indicated that the monolayer Ca_2_N might be mechanically exfoliated from the bulk, meanwhile, its electron confined layer could be maintained, and suitable encapsulation layers were proposed to protect it in ambient environment^19^.

Motivated by these recent experimental and theoretical progress on 2D electrides, and also by the surge of research activities on 2D materials in recent years initiated by the discovery of graphene, in this work, we conduct a systematically investigation of the electronic, dielectric, and plasmonic properties of monolayer and few-layer alkaline earth sub-nitrides Ca_2_N and Sr_2_N. We find that besides Ca_2_N, Sr_2_N is also dynamically stable down to monolayer thickness. Their phonon spectra exhibit characteristic features of 2D materials. For thickness greater than 1-ML, besides the two surface electron bands, there are additional bands crossing the Fermi energy which are due to the anionic electron layers confined in the 2D interlayer regions between the [X_2_N]^+^ layers. These interlayer bands are lower in energy than the surface bands and are nearly degenerate, indicating that they are only weakly coupled. The thickness dependence of properties such as surface energy, work function, and dielectric function are analyzed in detail. The change in property with thickness is most dramatic when going from 1-ML to 2-ML, which is associated with the appearance of the first interlayer electronic band. We find that Sr_2_N has a lower surface energy and lower work function compared with Ca_2_N. Due to their intrinsic structural anisotropy, these layered materials have highly anisotropic dielectric functions. In particular, the in-plane and out-of-plane components of dielectric function can have different signs, which occurs already for the bulk form in the near infrared frequency range. Moreover the dissipation is low in the range for the bulk materials. This shows that bulk Ca_2_N and Sr_2_N could be ideal low-loss indefinite media^20^. We further investigate the possibility of guided surface plasmon modes for these metallic materials and show that bulk Ca_2_N and Sr_2_N have good performance comparable to the noble metals but operating at lower frequencies, while their thin film structure can have strongly bounded plasmon modes which have advantage of much shorter wavelength compared to the conventional noble metals. Our findings thus identify a class of promising 2D electride materials and reveal their great potentials for future electronics and plasmonics applications.

## Results

### Crystal structure, dynamical stability, and surface energy

The alkaline earth subnitrides X_2_N (X = Ca, Sr, and Ba) in their bulk form can be synthesized by a direct solid-state reaction of the elements or through chemical reduction of the corresponding sesquinitrides^21^. Their structures consist of alternating ABC-stacked (X-N-X) hexagonal layers, which are in the 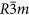 space group (anti-CdCl_2_-type) with a high *c*/*a* ratio, where *a* and *c* are the in-plane and out-of-plane unit cell dimensions^22^,^23^. Each (X-N-X) layer is closely packed. For example, the [Ca_2_N] layer has a thickness of 2.51 Å which is much smaller than that of the ordinary fcc Ca (111) layers (3.18 Å). This is typically attributed to the ionic bounding in the layer. Meanwhile, the separation between two neighboring layers is relatively large, leading to 2D confined spaces for the anionic electrons^15^.

**Figure 1:**
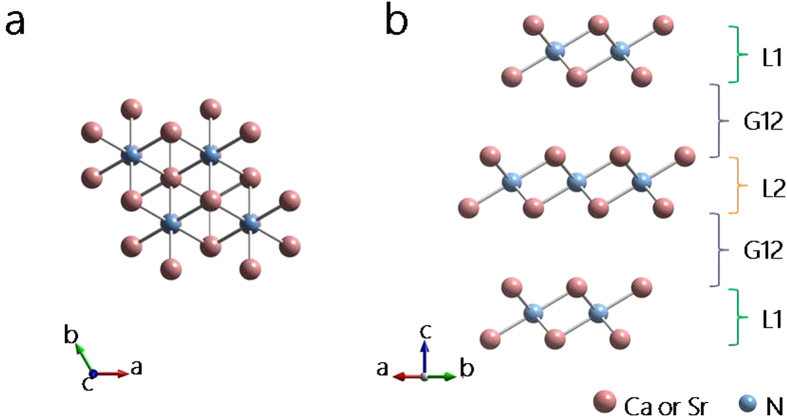
**a** Top and **b** side view of crystal structure of 3-ML X_2_N (X = Ca, Sr). It consists of three (X-N-X) unit layers with ABC-stacking. The symbols L1, L2, and G12 refer to the thicknesses of outermost layer, the next outermost layer, and the interlayer gap between L1 and L2, respectively, as explained in the caption of Tabel 1.

In this work, we investigate the properties of monolayer and few-layer thin films of these subnitrides (here one monolayer (ML) refers to one (X-N-X) unit layer). Since large interlayer separations (>3.5 Å) usually signal a possible role of long-range van der Waals interaction, the van der Waals corrections was included in the first-principles calculation^24^. The calculation details are described in the Method section. As an example, the crystal structure of 3-ML X_2_N (X = Ca, Sr) is schematically shown in Fig. 1. The optimized lattice parameters for isolated Ca_2_N and Sr_2_N thin films with thickness from 1-ML to 5-ML are listed in Table 1. (A comparison between DFT and experimental results for their bulk materials are shown in Supplementary Table S1, which exhibit good agreement between the two.) One observes that despite slight variations with respect to the number of layers, the calculated in-plane lattice constants, layer thicknesses, and interlayer distances are close to the experimental values of the corresponding bulk structures (for bulk Ca_2_N, *a* = 3.62 Å, layer thickness = 2.51 Å, and layer separation = 3.86 Å; for bulk Sr_2_N, *a* = 3.85 Å, layer thickness = 2.71 Å, and layer separation = 4.19 Å)^23^. Generally, the interlayer spacing is much larger than the layer thickness by about 50%. Sr_2_N has a larger interlayer spacing (around 4 Å) than Ca_2_N due to the larger atomic number of Sr.

**Figure 2:**
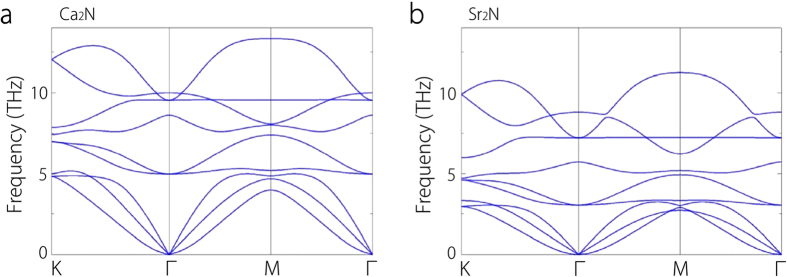
Phonon dispersions. **a**, Phonon dispersion of 1-ML Ca_2_N. **b**, Phonon dispersion of 1-ML Sr_2_N.

The dynamical stability of 1-ML Ca_2_N has been studied in Ref. 19. Here we find that few-layer Ca_2_N and Sr_2_N (including Sr_2_N monolayer) are also dynamically stable through analysis of their phonon spectra. In contrast, the similar structures of 1-ML and 2-ML Ba_2_N are not stable due to the presence of imaginary frequencies in their phonon spectra (see Supplementary Fig. S1 for the case of monolayer Ba_2_N). Therefore, we only focus on Ca_2_N and Sr_2_N in the present work. As representative examples, the phonon dispersions of monolayer Ca_2_N and Sr_2_N are shown in Fig. 2. The absence of imaginary frequencies in the whole Brillouin zone demonstrates the dynamical stability of the corresponding structures. The in-plane and out-of-plane transverse acoustic modes are not degenerate due to the structural anisotropy which is typical for layered materials. More importantly, while the in-plane transverse acoustic modes have a linearly dependence on their wavevectors in the vicinity of Γ-point, the out-of-plane acoustic (ZA) phonons exhibit a quadratic dispersion around Γ-point. The parabolic dispersion of ZA modes is a characteristic feature of layered materials, which is consistent with the macroscopic elastic theory of thin plates^25^. This feature has been frequently observed in other layered 2D materials, such as graphene^26^, layered transition metal dichalcogenides^27^, and phosphorene^28^.

**Figure 3:**
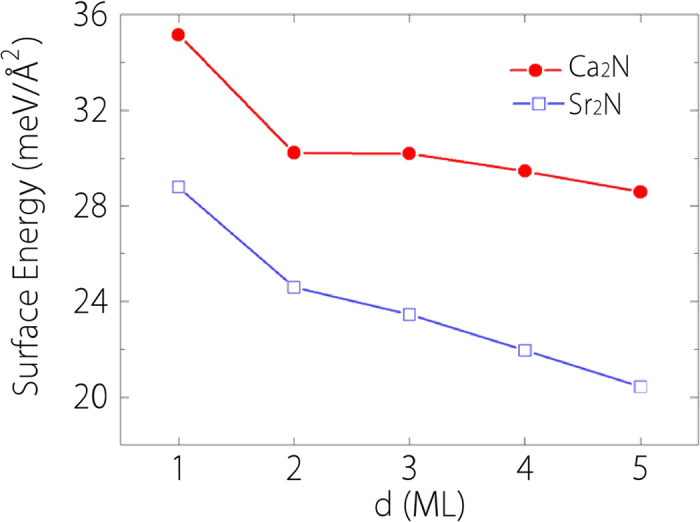
Surface energies for few-layer Ca_2_N and Sr_2_N as a function of thickness *d* from 1-ML to 5-ML.

We then calculate the surface energies of 1-ML to 5-ML Ca_2_N and Sr_2_N and the results are plotted in Fig. 3. Because of their layered structure and the large interlayer separations, the surface energies are relatively low. The calculated values are comparable to the surface energy of graphene (~12 meV/Å^2^)^29^. Previous calculations on 1-ML Ca_2_N have suggested its possibility to be mechanically exfoliated from the bulk. From our result in Fig. 3, the surface energy decreases with the increasing number of layers. Hence few-layer Ca_2_N would be more easily exfoliated compared with monolayer Ca_2_N, as naturally expected. Sr_2_N has an even larger interlayer spacing. Surface energy for 1-ML Sr_2_N is about 29 meV/Å^2^, and it decreases to about 20 meV/Å^2^ for 5-ML. With the same number of layers, Sr_2_N’s surface energy is smaller than that of Ca_2_N by about 6 ~ 8 meV/Å^2^. This implies that Sr_2_N monolayer and few-layers could be more easily obtained by mechanical exfoliation using Scotch tapes or AFM tips.

### Electronic structure and 2D confined electron layers

**Figure 4:**
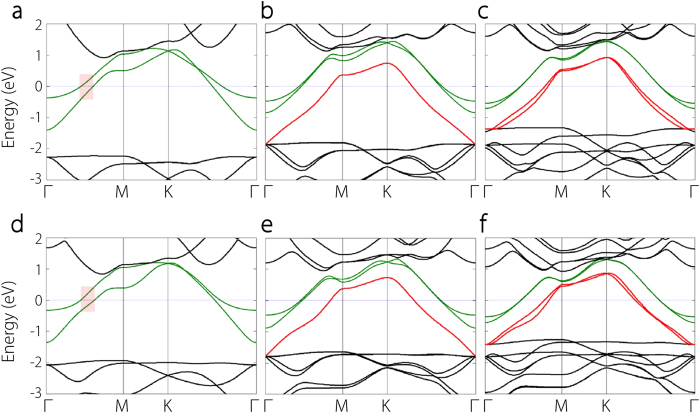
Electronic band structures of few-layer Ca_2_N and Sr_2_N. **a-c**, Band structure of Ca_2_N with **a** 1-ML, **b** 2-ML, and **c** 3-ML thickness. **d-f**, Band structure of Sr_2_N with **d** 1-ML, **e** 2-ML, and **f** 3-ML thickness. Fermi energy is set at zero. The two green colored bands are mainly from the 2D electron layers confined to the surface. The red colored bands (for films thicker than 1-ML) are mainly from the 2D electron layers confined in the interlayer regions. The red shaded rectangles in **a** and **d** indicate the regions contributing to the peaks ~0.3 eV in interband Im*ε*(*ω*) for 1-ML Ca_2_N and Sr_2_N.

Bulk Ca_2_N and Sr_2_N have conduction electrons confined in the 2D interlayer regions playing the role of anions^15^,^18^. One key question is whether these anionic electron layers would be maintained in their thin film structures. We find that the answer is positive. In Fig. 4, we show the electronic band structures of X_2_N (X = Ca, Sr) from 1-ML to 3-ML. First, one notices that all these systems are metallic, with Fermi level lying in partially filled dispersive energy bands. In 1-ML Ca_2_N (and Sr_2_N), there are two bands crossing Fermi level (see Fig. 4a,d). By inspecting the charge density distribution, one can verify that these two bands are from the 2D confined electron layers residing on the two sides of the [Ca_2_N]^+^ layer, and were referred to as the 2D electron gas in free space states^19^. The two bands are energy splitted due to the coupling between the two sides. When a second [Ca_2_N] layer is added, i.e. for a 2-ML structure, there appears an additional band crossing Fermi level, which has similar dispersion as the two surface bands but is lower in energy (see Fig. 4b). This band is from the confined anionic electron layer between the two [Ca_2_N]^+^ layers, as we will show in the following. The energy for this 2D band is lower because the confinement in the interlayer region is stronger than that for the surface, leading to a deeper potential well. One also observes that the splitting between the two surface bands is reduced, as a result of the reduced coupling between the two with increasing structural thickness. When going to 3-ML, there are two interlayer gap regions and indeed there is one more band crossing Fermi level (see Fig. 4c). One observes that the two bands from the interlayer-confined 2D electrons almost coincide, indicating that these states are strongly confined and the coupling between neighboring interlayer regions is very small. This clearly demonstrates the 2D character of the confinement topology. The above features persist when more [Ca_2_N] layers are added. Then more interlayer-confined anionic electron bands will appear and are nearly degenerate in energy. Their dispersion and bandwidth are almost independent on the thickness. For Sr_2_N, the band structure is quite similar to that of Ca_2_N with the same number of layers (with a slight decrease of the bandwidth, c.f. Fig. 4d–f), indicating that the 2D electride character is also maintained in its thin film form.

**Figure 5:**
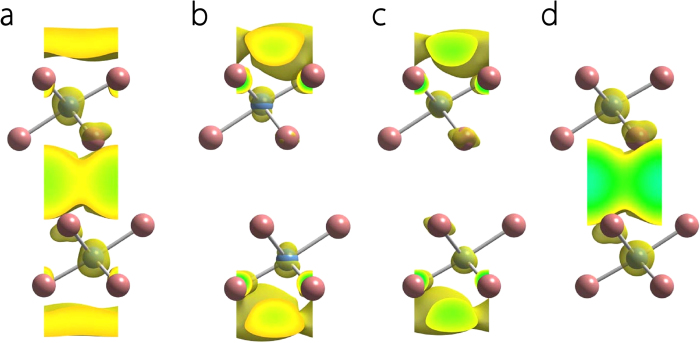
Electron density distribution in 2-ML Ca_2_N. **a**, Partial electron density isosurfaces (with value of 0.0003/Bohr^3^) for states in the energy range |*E* − *E*_*f*_| < 0.05 eV shown for a conventional unit cell. **b-d**, Band-decomposed electron density isosurfaces (with value of 0.003/Bohr^3^) for the three (colored) bands which cross Fermi level as shown in Fig. 4(b). **b** is for the upper green band, **c** is for the lower green band, and **d** is for the red band.

To visualize the real space distribution of the electronic states, we take 2-ML Ca_2_N as an example, and plot their partial electron densities for states within 0.05 eV around Fermi energy *E*_*F*_ in Fig. 5a. It is clear that the states around Fermi level are mainly located in three confined 2D regions: outside the two surfaces and in the interlayer space between the [Ca_2_N]^+^ layers. We further plot the total charge densities of each of the three conduction bands for 2-ML Ca_2_N. As shown in Fig. 5b–d, the lower band is from the 2D states in between the [Ca_2_N]^+^ layers, while the two higher bands are from the states confined on the surfaces. Similar result holds for Sr_2_N. This analysis validates our claims in the previous discussion.

Furthermore, electron-localization function (ELF) is useful for the analysis of the degree of electron localization and the bonding character^30^,^31^. From the ELF maps of 2-ML Ca_2_N and 2-ML Sr_2_N (see Supplementary Fig. S3), one finds that the bonding between the confined electron layers and the [Ca_2_N]^+^ ([Sr_2_N]^+^) layers is of ionic type. When one valence electron is removed, the surface confined layers will be vacated while the interlayer anionic electron layer remain largely intact (c.f. Supplementary Fig. S3).

**Figure 6:**
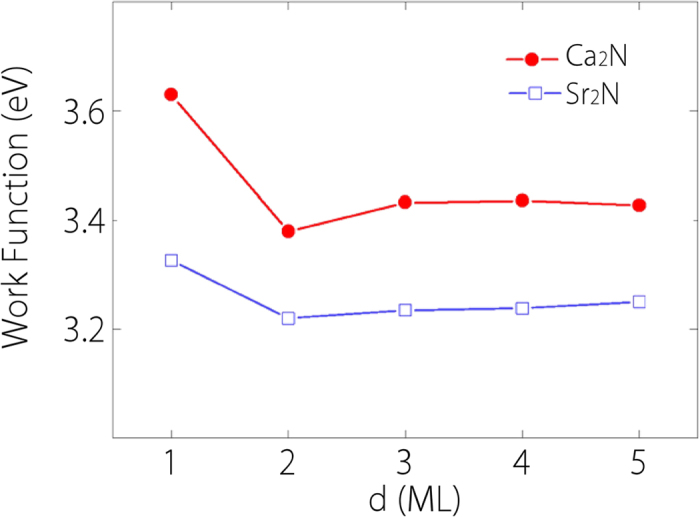
Work functions for few-layer Ca_2_N and Sr_2_N as a function of thickness *d* from 1-ML to 5-ML.

**Figure 7:**
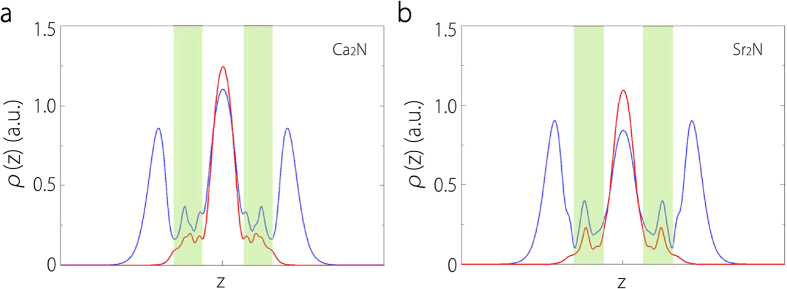
Partial density of states for the energy range |*E* − *E*_*f*_| < 0.05 eV averaged over the ab plane for **a** 2-ML Ca_2_N and **b** 2-ML Sr_2_N. In each figure, the blue curve and the red curve are for the distributions before and after one valence electron is removed, respectively. The green shaded regions indicate the locations of the [Ca_2_N]^+^ or [Sr_2_N]^+^ layers.

Previous studies found that bulk Ca_2_N has a highly anisotropic work function ranging from 2.6 eV (for (100) surface) to 3.4 eV (for (001) surface)^15^,^32^. In Fig. 6, we plot our results of the out-of-plane work function as a function of the number of layers for both Ca_2_N and Sr_2_N. One notes that there is a sharp decrease of the work function from 1-ML to 2-ML for both materials, due to the appearance of the strongly confined interlayer states that decouples the two surfaces. For Ca_2_N, with increasing layer number, the work function approaches a value around 3.4 eV, which is consistent with previous studies. The work function of Sr_2_N is less than that of Ca_2_N by about 0.2 eV, showing that the electrons in Sr_2_N are more loosely bound. We average the partial electron density around Fermi level in the layer plane. The resulting 1D density profiles for 2-ML with and without one electron removed are plotted in Fig. 7. One observes that there are three peaks corresponding to the three anionic electron layers. After one electron is taken away, the two side peaks disappear while the central peak remains. The two side peaks of Sr_2_N have larger width than those of Ca_2_N, implying a higher degree of delocalization of these surface states hence a lower work function.

### Dielectric function

The first-principles calculation based on DFT has proved to be a powerful tool for the study of dielectric function for metals, including ultrathin metallic films down to few-layer thickness^33^,^34^,^35^,^36^,^37^,^38^,^39^. In crystalline solids, the dielectric function *ε*(*ω*) consists of two contributions: a Drude-like intraband contribution and an interband contribution. The imaginary part of the interband contribution involves the interband matrix elements of the momentum operators, and can be evaluated directly in DFT^34^. Its real part can then be calculated via the Kramers-Kronig relation. The intraband contribution is typically treated by the Drude model, in which the plasma frequency (tensor) *ω*_*p*_ can be evaluated by DFT^34^. The calculation details are presented in the Method section.

**Figure 8:**
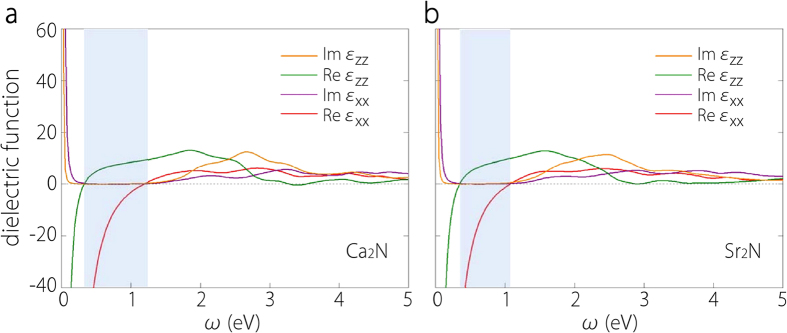
Anisotropic dielectric functions for bulk X_2_N: **a** for Ca_2_N and **b** for Sr_2_N. The real and imaginary parts of in-plane component *ε*_*xx*_ and out-of-plane component *ε*_*zz*_ are plotted using different colors. The blue shaded region in each figure indicates the frequency range in which Re*ε*_*xx*_ and Re*ε*_*zz*_ have different signs.

We first consider the dielectric properties of bulk Ca_2_N and Sr_2_N. Simple metals such as Au, Ag, and Al generally have a large isotropic bulk plasma frequency around 10 eV. In contrast, Ca_2_N and Sr_2_N are of layered structure, hence their dielectric properties are expected to exhibit intrinsic anisotropy. We find that bulk Ca_2_N has an in-plane (i.e. in the layer plane) plasma frequency *ω*_*p,xx*_ of 3.14 eV and an out-of-plane plasma frequency *ω*_*p,zz*_ of 0.95 eV; bulk Sr_2_N has an in-plane plasma frequency of 2.94 eV and an out-of-plane plasma frequency of 1.05 eV. These frequencies are significantly lower than those for simple metals, partly because the conducting electron density is lower. For each material, the out-of-plane plasma frequency is much lower compared with the in-plane plasma frequency, showing that the neighboring anionic electron layers are only weakly coupled. In Fig. 8, we plot the real and imaginary parts of in-plane and out-of-plane dielectric functions for the two materials. They exhibit the character of metallic behavior, i.e. Drude peaks at low energy due to intraband contribution and the real part of *ε*(*ω*) crossing from negative value to positive value with increasing frequency. The strong anisotropy in the dielectric function is also quite obvious. Remarkably, because of this anisotropy, the real part of in-plane dielectric function, Re*ε*_*xx*_(*ω*), changes sign at a frequency which is different from that for Re*ε*_*zz*_(*ω*), leading to an extended frequency window in which the two components have different signs (marked as the blue shaded regions in Fig. 8). This sign difference is the characteristic feature of so-called indefinite media^20^, which was proposed in the study of metamaterials and has important potential applications such as near-field focusing and building hyperlenses that can transform evanescent fields into propagating modes^40^,^41^. Previous realizations of indefinite media are mostly in artificially assembled structures which require complicated fabrication process and usually have high dissipation. Our results suggest that crystalline solids Ca_2_N and Sr_2_N in their bulk form would just be indefinite materials for a frequency range spanning the near infrared. Moreover, one notes that the imaginary parts of *ε*_*xx*_ and *ε*_*zz*_ in this frequency range are very small (~0.1), implying that they could be ideal low-loss indefinite materials.

**Figure 9:**
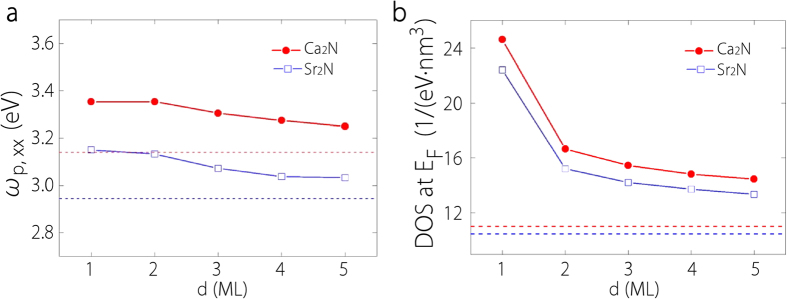
**a**, In-plane plasma frequency *ω*_*p*_,_*xx*_ for Ca_2_N and Sr_2_N thin films as a function of thickness *d* from 1-ML to 5-ML. **b**, electron density of states (DOS) of Ca_2_N and Sr_2_N at Fermi energy as a function of film thickness. In each figure, the dashed lines indicate the corresponding values for the bulk.

Next we focus on the dielectric functions of Ca_2_N and Sr_2_N few-layer structures. With decreasing film thickness, one expects that the anisotropy effects would be even stronger. The intraband contribution to the out-of-plane dielectric response *ε*_*zz*_ becomes negligible, and it has been shown that for simple metals which are isotropic in bulk can exhibit strongly anisotropic *ε*(*ω*) down to few-layer thickness^38^. In Fig. 9a, we plot the results of in-plane plasma frequency *ω*_*p*_,_*xx*_ as a function of the number of layers for both materials. One observes that the frequency slowly decreases with increasing number of layers and approaches its bulk value. The plasma frequency of Sr_2_N is lower than that of Ca_2_N by about 0.2 eV for each thickness. The square of plasma frequency is roughly proportional to the product of the carrier density and the inverse of effective mass. In Fig. 9b, one can see that from 1-ML to 2-ML, there is in fact a sharp decrease in the carrier density, as characterized by the DOS per unit volume around Fermi level, primarily due to the appearance of the large interlayer space of 2-ML. However, from the band structure in Fig. 4b, one notices that the additional 2D electron band (marked in red color) has a smaller effective mass around Fermi level than the two surface bands, hence decreasing the average effective mass. This compensates the decrease in carrier density, resulting in an overall small change in the plasma frequency between 1-ML and 2-ML.

**Figure 10:**
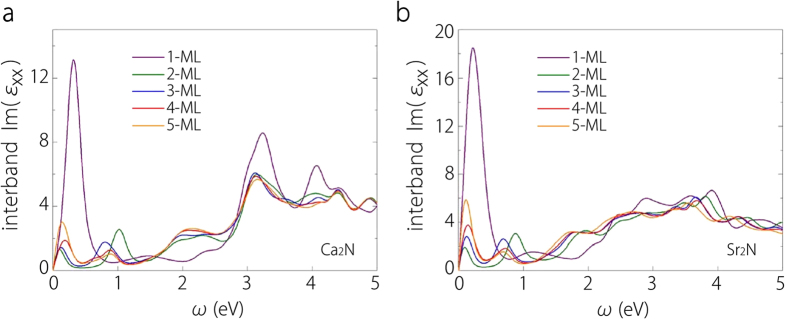
The interband contribution to Im*ε*_*xx*_(*ω*) for **a** Ca_2_N and **b** Sr_2_N thin film structures from 1-ML to 5-ML. Results for different thicknesses are plotted using different colors.

In Fig. 10, we show the interband contribution to the imaginary part of the in-plane dielectric function, Im*ε*_*xx*_(*ω*), which is closely connected to band structure features. One noticeable feature is the sharp peak around 0.3 eV for both materials at 1-ML thickness. This peak drops from 1-ML to 2-ML and its position shifts to a lower energy. Its appearance can be attributed to the (almost) parallel sections of the two surface bands for 1-ML, as indicated in the red shaded regions in Fig. 4a,d. From 1-ML to 2-ML, the splitting between two surface bands decreases and the volume increases a lot due to the appearance of the large interlayer region, resulting in the observed change of the peak. For thicknesses larger than 1-ML, there is another peak around 1 eV, which is due to the transition between the interlayer 2D bands and the surface bands (c.f. Fig. 4). From the band structures, it is clear that the response below 1eV is mainly contributed by the states from the 2D anionic electron layers.

The total in-plane and out-of-plane dielectric function with their real and imaginary parts for Ca_2_N few-layers are shown in Supplementary Fig. S4. For *ε*_*xx*_, the low energy part is dominated by the intraband Drude-like contribution. Re*ε*_*xx*_(*ω*) is negative below about 1eV. Meanwhile, the intraband contribution is negligible for *ε*_*zz*_(*ω*). Hence Re*ε*_*zz*_(*ω*) is largely positive for the low energy part. The sign difference between Re*ε*_*zz*_ and Re*ε*_*xx*_ in the low energy range again signals a possible indefinite material. Similar features are also demonstrated in the results for Sr_2_N few-layer structures (see Supplementary Fig. S5).

### Surface plasmon modes

Surface plasmon modes are confined electromagnetic excitations propagating at an interface between a conductor and a dielectric^42^. It typically requires a sign change of Re*ε* across the interface. Therefore the most commonly used materials for plasmonic applications are metals such as Au, Ag, and Al which have a range of frequencies with negative Re*ε*(*ω*)^43^. Since Ca_2_N and Sr_2_N are also metals, one may naturally wonder whether they could also support surface plasmon modes. This question is particularly interesting when considering the frequency range in which the material shows indefinite medium property. In such case, one expects that the positive Re*ε*_*zz*_ component may compete with the negative Re*ε*_*xx*_ component and tend to destroy the bounded plasmon modes.

Let’s first consider the interface between bulk Ca_2_N or Sr_2_N and a dielectric medium characterized by a frequency-independent dielectric constant *ε*_*d*_ > 0. The interface is parallel to the layer plane. As we have shown, Ca_2_N and Sr_2_N in the bulk form already have strong anisotropy in their dielectric functions. Assume that the interface supports a transverse magnetic (TM) plasmon mode travelling along the interface with a wave vector *β*. Following standard derivation using Maxwell’s equations^42^, one obtains that


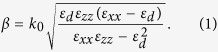


**Figure 11:**
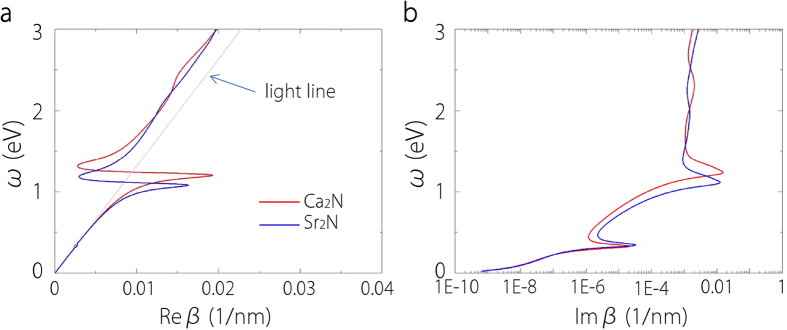
Dispersion relation of surface plasmon modes for an interface between a dielectric medium with *ε*_*d*_ = 2.25 and bulk X_2_N (X = Ca, Sr). The light line in the dielectric medium is also shown in grey color.

Here *k*_0_ = *ω*/*c* is the wave vector in vacuum, *c* is the speed of light, and *ε*_*xx*_ and *ε*_*zz*_ are the two components of the dielectric function for our conducting material. If *ε*_*xx*_ = *ε*_*zz*_, the result in Eq.(1) reduces to the familiar result for isotropic metal^42^. The dispersion characteristics of the surface plasmon modes for both materials are shown in Fig. 11, with *ε*_*d*_ = 2.25 (appropriate for SiO_2_). In Fig. 11a, the modes lying to the right of the light line (in the dielectric medium) are bounded to the surface. One observes that the results show characteristic surface plasmon peaks similar to simple metals and confined plasmon modes still exist within the frequency range where the material shows indefinite medium property. A major difference is that for simple metals the peak position, known as surface plasmon frequency *ω*_sp_, occurs at higher energies, e.g. *ω*_sp_ ~ 3.4 eV for Ag/SiO_2_ interface^44^; while *ω*_sp_ here is much lower, around 1.1 eV–1.2 eV in the near infrared range. The Im*β* shown in Fig. 11b is connected to the energy damping during the mode propagation. Since high dissipation occurs around *ω*_sp_, for practical applications, modes with frequencies less than *ω*_sp_ are used. Here for Ca_2_N, if we take *ω* = 1.02 eV, the corresponding surface plasmon wavelength λ_sp_ ≃ 656 nm, and we have a long propagation length *L* = 1/(2Im*β*) ≃ 5.15 *μ*m and the decay length in the dielectric is 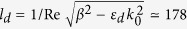 nm. Longer propagation length can be achieved at lower frequencies, e.g. at *ω* = 0.64 eV, we can have *L* ≃ 173 *μ*m but the mode confinement is reduced, with *l*_*d*_ ≃ 737 nm. These values are comparable to those for the noble metals (in the visible or UV frequency range) which are the usual building blocks for plasmonic devices. However, the operating frequency here is much lower. From above discussion, we see that despite the intrinsic anisotropy, bulk Ca_2_N and Sr_2_N can still support surface plasmons and could be suitable plasmonic materials in the near infrared frequency range. Experimentally, these surface plasmon modes may be excited, e.g. by prism coupling or by near-field excitation, and probed by standard optical techniques such as near-field optical microscopy, leakage radiation imaging, or scattered light imaging^42^.

**Figure 12:**
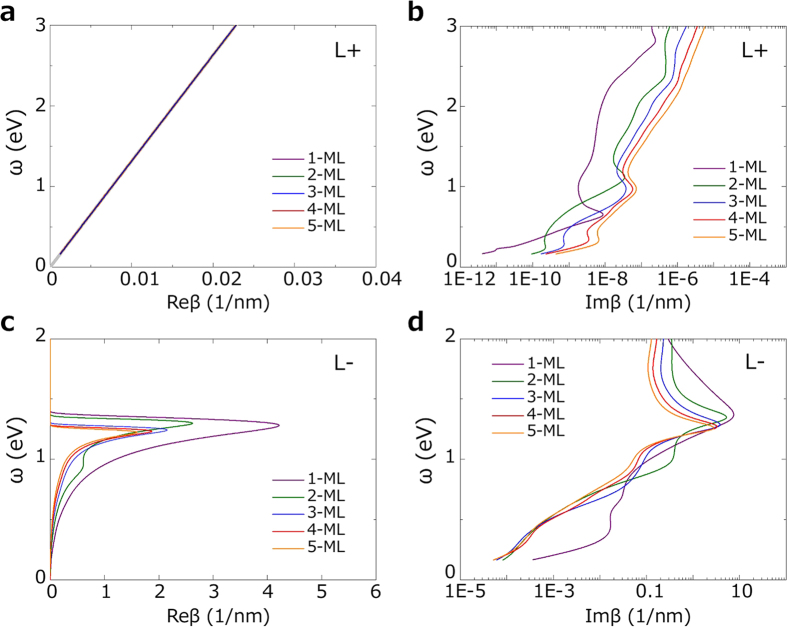
Dispersion characteristics of surface plasmon modes for Ca_2_N few-layers in a dielectric medium with *ε*_*d*_ = 2.25. **a** and **b** are for the antisymmetric mode (*L*+ mode). **c** and **d** are for the symmetric mode (*L*− mode). **a** and **c** show the real part of the wave number component. **b** and **d** show the imaginary part of the wave number component.

We then turn to the thin films of Ca_2_N and Sr_2_N sandwiched between dielectric materials. Besides the change in the thickness-dependent dielectric function, an important effect in thin films is that the surface plasmon modes at two interfaces could couple and form two modes with opposite parity: a symmetric mode (*L*−) and an antisymmetric mode (*L*+)^44^. Their dispersions have been derived before in the study of ultrathin metallic films^38^, and are quoted in our Method section. In Fig. 12, we plot the surface plasmon dispersion characteristics for Ca_2_N with different film thicknesses. One observes that the antisymmetric modes are lying on the light line of the dielectric medium, indicating that they are squeezed out of the metal region forming unbounded modes propagating in the dielectric material, which is similar to the case of Au ultrathin films. Meanwhile, pronounced plasmonic peaks do show up for the symmetric modes, clearly indicating that these are bounded surface plasmon modes. Again, one observes that the variation with thickness is most dramatic between 1-ML and 2-ML. For 1-ML, the bounded modes occur around 1 eV. The high peak in Re*β* vs. *ω* shows the modes are strongly bounded to the metallic layer. For thicknesses of 2-ML to 5-ML, the dispersions are quite close. The corresponding surface plasmon frequencies shift to around 1.2 eV. As for the imaginary part of *β*, 1-ML structure has relatively large values, whereas Im*β* for 2-ML to 5-ML almost collapse on a single curve and deceases rapidly in the range below 0.8 eV. For Sr_2_N few-layers, the results for surface plasmon dispersion show similar features. The dispersions for symmetric (*L*−) modes are shown in Supplementary Fig. S6. The antisymmetric (*L*+) modes are again unbounded hence are not shown.

**Figure 13:**
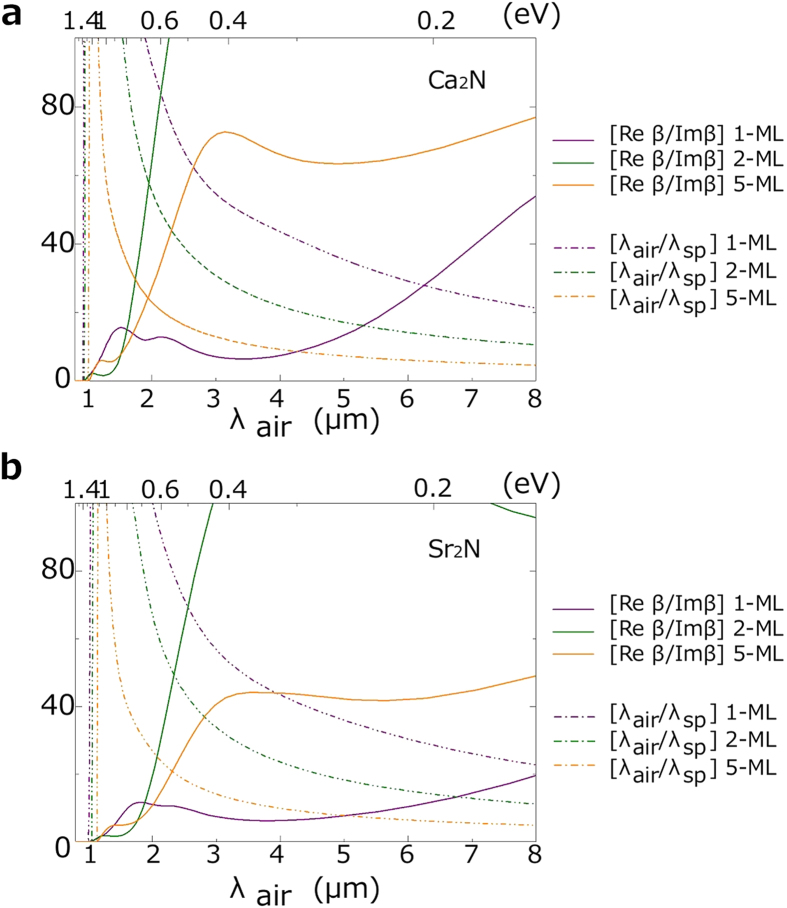
Characteristics Re*β*/Im*β* and λ_air_/λ_sp_ for the symmetric surface plasmon modes in few-layer **a** Ca_2_N and **b** Sr_2_N, plotted as functions of the wavelength in air λ_air_. λ_sp_ is the surface plasmon wavelength. The dielectric medium is with *ε*_*d*_ = 2.25.

Compared with their bulk results, one observes that the surface plasmon frequency *ω*_sp_ is more or less the same, but both Re*β* and Im*β* are greatly increased by two orders of magnitude. This means that the plasmon wavelength and the confinement scale are much smaller, which are desired features. However, the propagation length is also decreased at the same time. Therefore, it is more meaningful to consider the dimensionless ratio Re*β*/Im*β* (which measures how many surface plasmon wavelengths can be covered before the wave loses most of its energy) as well as the wave localization (or wave shrinkage) quantified by λ_air_/λ_sp_, where λ_air_ = 2*πc*/*ω* is the wavelength in air. In Fig. 13, we show these two dimensionless characteristics for the two materials as functions of λ_air_. The results for 3-ML and 4-ML are similar to those of 5-ML hence are not shown. One observes that for 1-ML Re*β*/Im*β* is small for most frequencies, due to the relatively high dissipation associated with Im*β* (c.f. Fig. 12d). Generally, the wave localization reaches its peak near the surface plasmon resonance. However, there the ratio Re*β*/Im*β* is small due to the enhanced dissipation. While larger Re*β*/Im*β* can be achieved for longer wavelengths, the wave localization becomes poor in that range. This tradeoff is a typical feature for surface plasmons. For application purposes, a compromise may be reached somewhere in between when the two are comparable.

Since 1-ML Ca_2_N and Sr_2_N have considerably larger dissipation, in the following we take 2-ML Ca_2_N and Sr_2_N as examples. For λ_air_ = 2 *μ*m (0.64 eV) in the near infrared range, 2-ML Ca_2_N has a decay length *l*_*d*_ = 5.4 nm in the dielectric, showing that the plasmon mode is strongly confined to the metallic layer. The ratios λ_air_/λ_sp_ = 56 and Re*β*/Im*β* = 61. These two values are considerably larger than what noble metals could achieve at their optimal working frequencies. The corresponding values for Sr_2_N at λ_air_ = 2 *μ*m (*ω* = 0.64 eV) are given by *l*_*d*_ = 4.4 nm, λ_air_/λ_sp_ = 68 and Re*β*/Im*β* = 17. In comparison, the surface plasmon modes for noble metals such as Au and Ag are unbounded at this wavelength. Noticeably, the surface plasmon wavelengths in these materials are very small, λ_sp_ = 34.9 nm for Ca_2_N and λ_sp_ = 28.9 nm for Sr_2_N (at *ω* = 0.64 eV), while the shortest λ_sp_ for Au and Ag (occur in the visible light range) would be at least larger than 100 nm. These suggest that Ca_2_N and Sr_2_N thin films have great potential for making plasmonic devices operating in the near infrared range with much smaller scales.

## Discussion

From our previous analysis of the electronic structures of these electride materials, one expects that the loosely bound surface anionic electrons are highly reactive in ambient conditions. They may act as good electron donors and as excellent catalysts for chemical reactions^11^. For physical applications, one needs to stabilize its property, e.g. by effective encapsulation. For 1-ML Ca_2_N, a possible encapsulation scheme using 2D insulating layers of graphane was proposed^19^. In the case of few-layer alkaline earth subnitrides, since as we discussed each interlayer anionic electron layer are strongly confined in 2D regions and the coupling between them is small, one expects that the most reactive electrons are from the surface layers, while the electron layers inside should be less reactive. A detailed study of this point and possible encapsulation schemes for Sr_2_N will be deferred to a future work.

For electride materials, the topology of the cavities confining anionic electrons is one key factor determining their properties. Although the cavity topologies for both Ca_2_N and Sr_2_N are similar, compared with Ca_2_N, Sr_2_N has a smaller electrostatic potential associated with the larger atomic number of Sr. This was reflected in its lower work function, and may also lead to a high electron mobility for Sr_2_N few-layers^18^. Previous experimental studies have shown that bulk Ca_2_N has high mobility of 520 cm^2^/(V·s)^15^. One expects that the mobility for Sr_2_N may be even higher. Hence Sr_2_N few-layers as new 2D conducting materials could have a good potential for electronics applications.

As having been demonstrated for other 2D layered materials, strain engineering has proved to be a powerful tool to modify and control the material properties. The layered materials discussed here have a large interlayer spacing (>3.5 Å) between the [X_2_N]^+^ layers. We also expect that applying strain could be a good method to tune the material properties such as the dielectric function, the plasmonic dispersion, and the carrier mobility. A systematic study of the strain effects is currently underway.

## Methods

### First-principles calculations

First-principles calculations are carried out using the Vienna *ab*-initio simulation package (VASP)^45^,^46^, based on the density functional theory (DFT). The exchange-correlation functional is treated using Perdew-Burke-Ernzerhof generalized gradient approximation^47^. The projector augmented wave (PAW) method^48^ is employed to model interactions between electrons and ions. The treated valence electrons are the 3p4s, 4s4p5s, and 2s2p for the Ca, Sr, and N atoms, respectively. The cutoff for plane-wave expansion is set to be 600 eV. The vertical distance between thin films (the thickness of the vacuum gap) is at least 18 Å, which is large enough to avoid artificial interactions between the film and its periodic images. Both the atomic positions and lattice constant were fully relaxed using conjugate gradient method. The convergence criteria for energy and force were set to be 10^−5^ eV and 0.01 eV/Å, respectively. DFT-D2 method was applied to describe the long-range van der Waals interaction. The Brillouin zone integrations have been carried out on a Γ-centered *k*-mesh. Monkhorst-Pack k-point meshes^49^ with sizes of 15 × 15 × 1 and 31 × 31 × 1 were used for geometry optimization and static electronic structure calculation, respectively. In the later study of optical properties, the sizes of *k*-mesh are significantly increased to 61 × 61 × 1 and 41 × 41 × 9 for thin films and bulk respectively, to achieve highly converged results. For the integration over the Brillouin zone in calculating dielectric functions, we used the first order Methfessel-Paxton method^50^ with a value of 0.1 eV. The phonon dispersions of the structures were calculated by using density functional perturbation theory as implemented in the PHONOPY code^51^,^52^.

### Calculation of dielectric functions

The optical properties of solids are mainly due to the response of the electron system to a time-dependent electromagnetic perturbation. For metals, the optical complex dielectric function consists of interband and Drude-like intraband contributions:


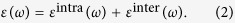


The imaginary part of the interband part can be calculated using the results from DFT calculations as^34^


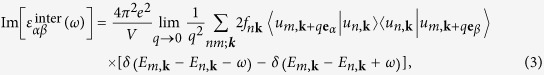


where *α* and *β* refer to Cartesian coordinates, **e**_*α*(*β*)_ are unit vectors, *V* is the volume of the unit cell, 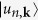 and *E*_*n*,**k**_ are the periodic part of the Bloch wave function and the corresponding eigenenergy for band *n* and wave vector **k**, and *f*_*n***k**_ is the Fermi-Dirac distribution function. The real part of interband contribution can be obtained through the Kramers-Kronig relation. The intraband contribution is usually modeled by the Drude model:


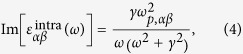



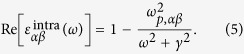


Here *γ* is a life-time broadening obtained either from a higher-order calculation or from experiments. In our calculation, we used the experimental determined electron life-time (of 0.6 ps)^15^ for bulk Ca_2_N to estimate *γ* (~1.1 meV). The same value was also used for the calculations of Sr_2_N due to their similar electronic structures. We have checked the sensitivity of our results’ dependence on *γ* by repeating the calculations of *ε*(*ω*) with *γ* value varying from *γ*/10 to 5*γ*. The obtained results are of little difference. This is because the value of *γ* is already quite small (reflecting the fact that these materials are good metals). The *ω*_*p,αβ*_ is the plasma frequency tensor which can be calculated using


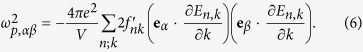


The dielectric functions and plasma frequencies were suitably renormalized to exclude the vacuum region from the unit cell in our calculations.

### Surface plasmon modes calculations

For thin film structures, the plasmon modes at two surfaces would couple and form two modes with different parity^44^. The equations governing their dispersions have been derived before^38^:


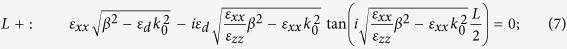



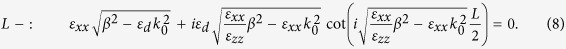


The first equation above is for the antisymmetric (*L*+) mode and the second equation is for the symmetric (*L−*) mode. Here *L* in the equations is the film thickness. Other quantities in these two equations are defined in the main text. In our calculation, we solve the two equations numerically using a two-dimensional unconstrained Nelder-Mead minimization algorithm^53^ with a tolerance of 10^−13^ nm^−1^ in the complex wave vectors.

## Additional Information

**How to cite this article**: Guan, S. *et al*. Electronic, Dielectric, and Plasmonic Properties of Two-Dimensional Electride Materials X_2_N (X=Ca, Sr): A First-Principles Study. *Sci. Rep*. **5**, 12285; doi: 10.1038/srep12285 (2015).

## Supplementary Material

Supplementary Information

## Figures and Tables

**Table 1:** Lattice parameters of Ca_2_N and Sr_2_N thin films with thicknesses from 1-ML to 5-ML.

No. of Layers	*a* (Å)	*t* (Å)	L1 (Å)	L2 (Å)	L3 (Å)	G12 (Å)	G23 (Å)
Ca_2_N							
1	3.562	2.516	2.516				
2	3.562	8.608	2.509			3.598	
3	3.552	14.957	2.514	2.504		3.712	
4	3.550	20.099	2.516	2.507		3.721	3.793
5	3.555	26.419	2.513	2.505	2.508	3.703	3.823
Sr_2_N							
1	3.778	2.761	2.761				
2	3.775	9.427	2.757			3.913	
3	3.770	16.260	2.760	2.748		3.983	
4	3.765	21.821	2.764	2.754		4.016	4.068
5	3.768	28.562	2.758	2.751	2.753	3.989	4.061

Here *a* is the in-plane lattice constant, *t* is the thickness of the film (vertical distance between the top and bottom atomic layers), L(1, 2, 3) refer to the thicknesses of the [X_2_N]^+^ layers, L1 is for the outermost layer(s), L2 is for the next outermost layer(s) and so on (see Fig. 1), and G*ij* refers to the thickness of the interlayer gap region between Layer *i* and Layer *j*.
