# Supplementary material for: Electronic, Dielectric, and Plasmonic Properties of Two-Dimensional Electride Materials X2N (X=Ca, Sr): A First-Principles Study
Source: Sci Rep. 2015 Jul 20;5:12285. doi: 10.1038/srep12285 (PMC5387395; doi:10.1038/srep12285)
Supplement: Supplementary Information [file srep12285-s1.pdf]

Supplementary Information

Electronic, Dielectric, and Plasmonic Properties of Two-Dimensional Electride Materials X_2N (X=Ca, Sr): A First-Principles Study.

Shan Guan[1,2], Shengyuan A. Yang[2,⋆], Liyan Zhu[3], Junping Hu[1], Yugui Yao[1,⋆]

[1] *School of Physics, Beijing Institute of Technology, Beijing 100081, China*

[2] *Research Laboratory for Quantum Materials and EPD Pillar, Singapore University of Technology and Design, Singapore 487372, Singapore*

[3] *School of Physics and Electronic & Electrical Engineering, Huaiyin Normal University, Huaian 223300, China*

⋆ *Corresponding authors.*

Shengyuan A. Yang

Tel: +65-64994785

e-mail: shengyuan_yang@sutd.edu.sg

Tel: +86-10-68918672

Yugui Yao

e-mail: ygyao@bit.edu.cn

Supplementary Contents

Supplementary Fig. S1

As shown in Fig.S1, the corresponding monolayer structure of Ba_2N is not dynamically stable due to the presence of imaginary frequencies in the phonon spectrum.

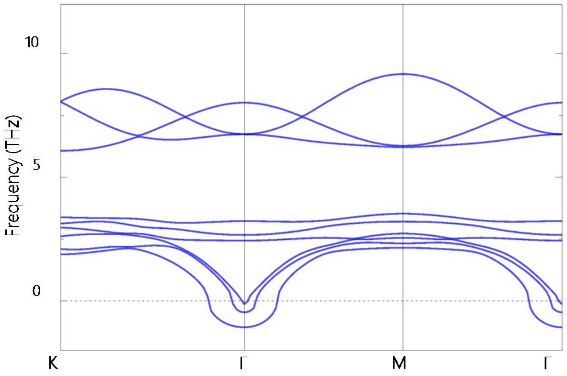

Supplementary Fig. S1. Phonon dispersion of 1-ML Ba_2N.

Supplementary Fig. S2

The stability of 1-ML Ca_2N was assessed and confirmed by molecular dynamics (MD) simulation in Ref.19 (of the main text). Here we carry out similar MD simulation for 1-ML Sr_2N at 300 K using VASP. A 3×3 supercell is used in the simulation. After running 3000 steps with a time step of 1 fs, no bond is broken, suggesting that the structure of Sr_2N monolayer is stable. Fig. S2 shows a snapshot of the structure after 3 ps simulation time.

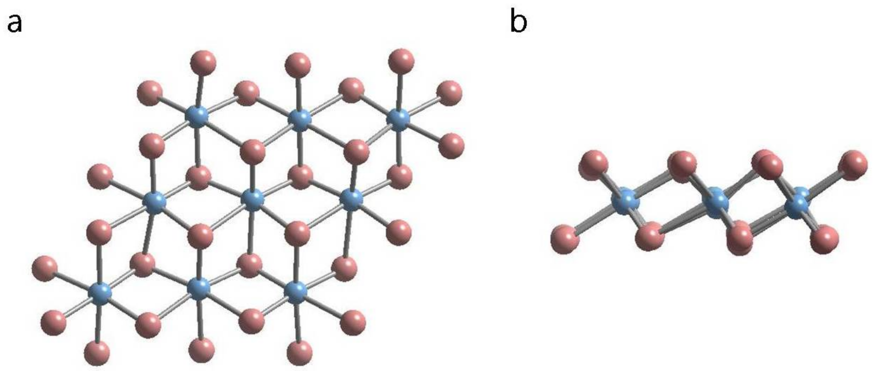

Supplementary Fig. S2. **a** Top and **b** side views of 1-ML Sr_2N after 3 ps MD simulation time.

Supplementary Fig. S3

In Fig.S3a and Fig.S3c, we show the ELF maps of 2-ML Ca_2N and 2-ML Sr_2N. Three delocalized electron layers can be seen. One observes that there is no bonding localization attractor between the confined electron layers and the $[Ca_2N]^+$ ($[Sr_2N]^+$) layers, indicating the bonding between them is of ionic type. When one valence electron is removed, the surface confined layers will be vacated while the interlayer anionic electron layer remain largely intact, as shown in Fig.S3b and Fig.S3d. This feature also implies that the out-of-plane work function of these layered materials are mainly determined by the 2D electron layers on the surface.

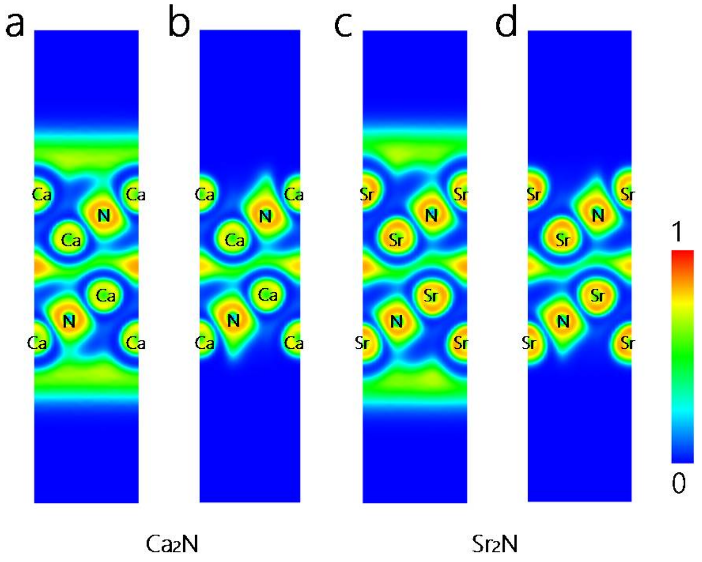

Supplementary Fig. S3. **Electron localization function (ELF) maps of the (-1 2 0) plane for 2-ML Ca_2N and Sr_2N**. **a** and **c** are the ELF maps for Ca_2N and Sr_2N respectively, shown for a conventional unit cell. **b** and **d** are for $[Ca_2N]^+$ and $[Sr_2N]^+$ respectively, where one valence electron is removed.

Supplementary Fig. S4 and Fig. S5

The total in-plane and out-of-plane dielectric function with their real and imaginary parts for Ca_2N few-layers are plotted in Fig.S4. The sign difference between Reε_{zz} and Reε_{xx} in the low energy range again signals a possible indefinite material. One observes that the change of $\varepsilon(\omega)$ against thickness is most dramatic from 1-ML to 2-ML, due to the introduction of the first interlayer anionic electron layer. The imaginary part of dielectric function for 1-ML is much larger than that for 2-ML, particularly in the near infrared frequency range, which indicates more energy dissipation for possible plasmon modes as we discuss in the main text. In addition, the variation of ε_{xx} versus thickness is overall less dramatic compared with that of ε_{zz}, which again reflects the fact

that the conducting electrons are strongly confined within the 2D in-plane layered regions. Due to the same reason that the different conducting 2D layers are only weakly coupled, the results for both in-plane and out-of-plane components converges rapidly with increasing thickness (note that the low energy part of the out-of-plane component would eventually be dominated by a Drude-like intraband contribution when approaching the bulk limit). One notes that there is only small difference between the results of 4-ML and 5-ML. We also calculated 8-ML which is the largest system size within our computing capability. The obtained results are very close to those for 5-ML. Similar features are also demonstrated in the results for Sr_2N few-layer structures, as shown in Fig.S5.

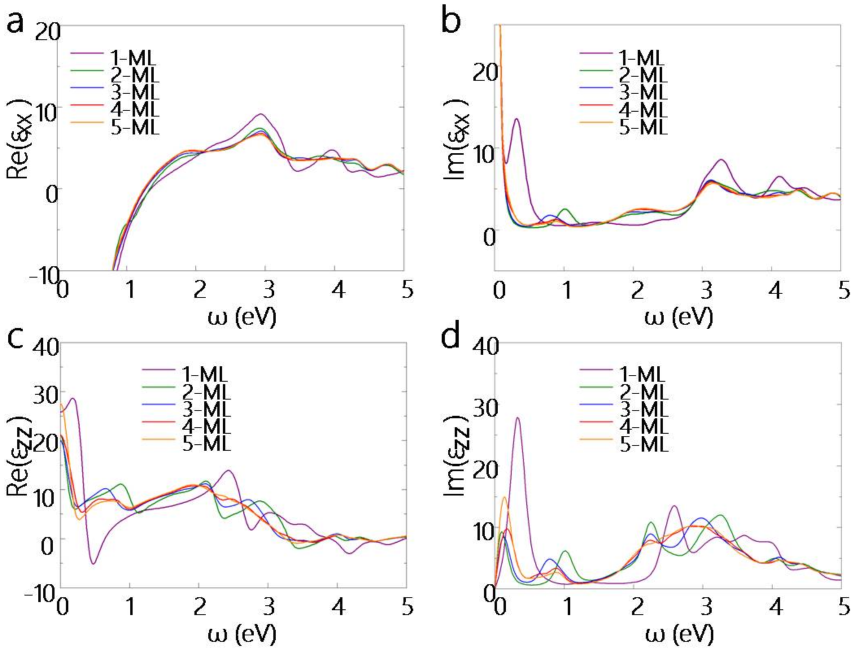

Supplementary Fig. S4. **Dielectric functions for Ca_2N few-layers.** **a** and **b** are for the real and imaginary parts of in-plane component ε_{xx} respectively. **c** and **d** are for the real and imaginary parts of out-of-plane component ε_{zz} respectively. Results for different thicknesses are plotted using different colors.

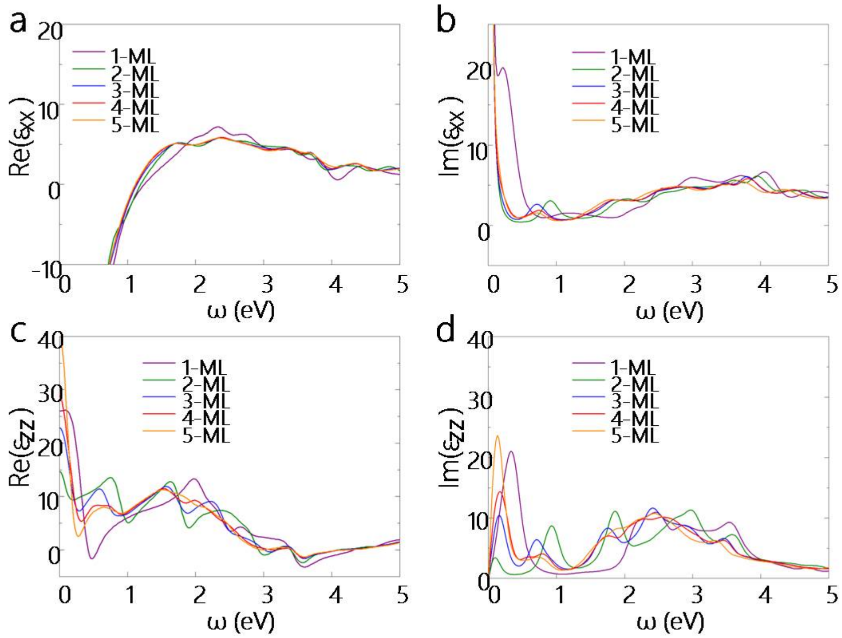

Supplementary Fig. S5. **Dielectric functions for Sr_2N few-layers.** **a** and **b** are for the real and imaginary parts of in-plane component ε_{xx} respectively. **c** and **d** are for the real and imaginary parts of out-of-plane component ε_{zz} respectively. Results for different thicknesses are plotted using different colors.

Supplementary Fig. S6

The dispersions for symmetric ($L-$) surface plasmon modes of Sr_2N few-layers are plotted in Fig.S6, which show similar features as the case of Ca_2N few-layers (c.f. Fig.12 of the main text).

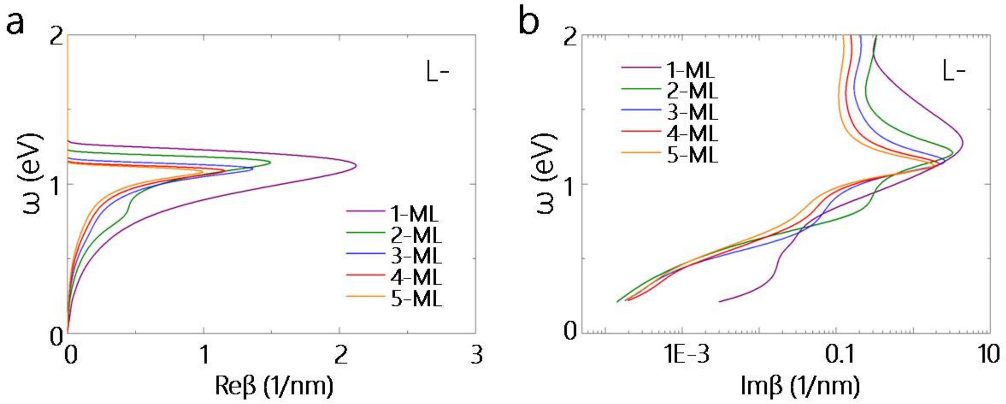

Supplementary Fig. S6. Dispersion characteristics of $L-$ symmetric surface plasmon modes for Sr_2N few-layers in a dielectric medium with $\varepsilon_d = 2.25$. **a** shows the real part of the wave number component. **b** shows the imaginary part of the wave number component.

Supplementary Table. S1

Structure parameters obtained from first principles calculations compared with experimental data for bulk Ca_2N and Sr_2N. Here a is the in-plane lattice constant, c is vertical lattice constant of a unit cell, L refers to the thickness of the $[X_2N]^+$ layers, and G refers to the thickness of the interlayer gap region between the $[X_2N]^+$ layers.

	a (Å)	c (Å)	L (Å)	G (Å)
Ca_2N				
Experiment	3.623	19.101	2.511	3.856
DFT	3.595	18.892	2.508	3.789
Sr_2N				
Experiment	3.852	20.688	2.712	4.185
DFT	3.796	20.505	2.734	4.111
